# Objectively measured sedentary time and physical activity time across the lifespan: a cross-sectional study in four age groups

**DOI:** 10.1186/1479-5868-9-149

**Published:** 2012-12-18

**Authors:** Heleen Spittaels, Eveline Van Cauwenberghe, Vera Verbestel, Femke De Meester, Delfien Van Dyck, Maïté Verloigne, Leen Haerens, Benedicte Deforche, Greet Cardon, Ilse De Bourdeaudhuij

**Affiliations:** 1 Department of Movement and Sport Sciences, Faculty of Medicine and Health Sciences, Ghent University, Watersportlaan 2, Gent, 9000, BELGIUM; 2 VUB, Brussels, Belgium; 3 Research Foundation Flanders, Brussels, Belgium

**Keywords:** Accelerometer, Light-intensity physical activity, Objective assessment, Sitting time

## Abstract

**Background:**

From a health perspective it is suggested to promote a positive balance between time spent in light intensity physical activity (LIPA) and sedentary behaviour (SB) (i.e. spending more time in LIPA than time spent in SB). However, no studies have reported prevalence rates of the LIPA-SB balance yet. The aim of this study was to objectively investigate the time spent in SB, in LIPA and moderate-to-vigorous intensity physical activity (MVPA) in four Belgian age groups and to explore which proportion of the population had a favorable balance between LIPA and SB and combined this with recommended amount of MVPA.

**Methods:**

Accelerometer data from 7 cross-sectional studies (N=2083) in four age groups (preschoolers, primary schoolchildren, secondary schoolchildren and adults) were aggregated. Differences in SB and PA between age groups and between men and women were determined by two-way MANCOVA. LIPA-SB balance was calculated and participants were categorized into one of four groups: (1) positive LIPA-SB balance (LIPA> SB) & sufficient MVPA (2) negative LIPA-SB balance & sufficient MVPA (3) positive LIPA-SB balance & insufficient MVPA (4) negative LIPA-SB balance & insufficient MVPA.

**Results:**

For the total sample, 55% of the waking time was spent in SB, 39% in LIPA and 6% in MVPA. Differences in SB between age groups was dependent from gender (p<0.001). Further, a positive LIPA-SB balance was assessed in 18% of the total sample and only 10% combined this positive balance with recommended amount of MVPA. Secondary schoolgirls were most at risk, with only 1% of the sample combining a positive LIPA-SB balance with sufficient MVPA. Another risk group was the large proportion (43%) of adult men who combined sufficient MVPA with a negative LIPA-SB balance.

**Conclusion:**

A high proportion of the Belgian population is at risk if taking into account both SB and PA levels. Secondary schoolgirls have the unhealthiest SB and PA profile and are therefore an important target group for interventions both increasing MVPA and decreasing SB. In men more attention should be given in promoting a positive LIPA-SB balance independently from their compliance with the MVPA guidelines.

## Background

For many years, epidemiological evidence has demonstrated the positive health outcomes of regular moderate-to-vigorous intensity physical activity (MVPA) in all age groups [1], [2-4], [5]. Recently, there has been emerging evidence suggesting that next to MVPA, sedentary behavior (SB) has an important independent influence on health. The Sedentary Behavior Research Network [6] defines SB as any waking behaviour characterised by an energy expenditure of ≤ 1.5 METs while in sitting or reclining posture. According to this definition, SB is not necessarily the same as a lack of regular MVPA. If an adult person walks 30 minutes at moderate intensity daily, he/she fulfils the current PA recommendation [7]. But at the same time, if he/she has a sedentary job, he/she is likely to be sitting for the rest of the day. Although Morris et al. [8] already identified the negative health effects of occupational sitting in the 1950s, only recently an increasing number of adult studies has indicated that both total sitting time and prolonged periods of sitting time exert important negative health effects independently of the positive effects of sufficient engagement in PA [9], [10], [11]. In adults, too much sitting has been independently associated with abnormal glucose tolerance [9,12], metabolic syndrome [13], [14], type 2 diabetes [15], cardiovascular risk factors [16,17] and endometrial cancer [18] . In youth, the relationship between SB and health is still unclear. Some studies found associations between objectively measured sedentary time and adiposity and an adverse cardiometabolic risk profile [19]; [20] while others did not [21]. However, evidence in youth regarding the positive relationship between screen time behaviour and negative health outcomes is found. Recently, two reviews have been published [22], [23] and both concluded that there was evidence for a negative effect of screen time on young peoples’ aerobic fitness. Further, results of Tremblay et al. [23] showed that sedentary time, in the majority of the studies assessed by TV viewing time, also has a negative impact on young people’s body mass index (BMI), cardiovascular health, self-esteem, pro-social behavior and academic achievement.

As SB has been put forward as an independent risk factor for several health problems, it is crucial to investigate the quantity of SB in the population. According to Owen et al. [24], measuring prevalence rates of SB in populations is one of the phases in the behavioral epidemiology framework [25,26] with only modest evidence for a population health science of SB and more accurate measurements are recommended. However most of the published studies investigating SB used self-report methods and focused only on television viewing [27],while it is advocated to assess a wide range of sedentary activities [1] and to preferentially use objective measurement methods instead of subjective methods [28]. Until now, large population studies, using objective measurements to assess SB prevalence rates across the lifespan are scarce. Matthews et al. [29] used accelerometer data of the National Health and Nutrition Examination Survey (NHANES) to assess SB in a representative US sample of 6–85 years old and found that Americans spent 55% of their waking hours sedentary. Other studies focused mostly on narrower age ranges. In adults, percentages found for time spent in SB were very similar in Sweden (55% of waking hours) [30]; US (57%) [31] and Australia (57%) [32]. In the European Youth Heart Study, children’s prevalence rates of SB ranged between 42 % (9 year-olds) and 58% (15 year-olds) [33]. These percentages demonstrate that both children and adults in Western countries spent most of their waking hours in SB.

From a health perspective it is important to minimize the time spent in SB. However, a large proportion of the population will not consider replacing their sitting time with physical activities at moderate to vigorous intensity (MVPA). An alternative is to substitute sedentary time with light-intensity physical activities (LIPA) e.g. standing, stretching or walking to another room. As Healy et al. [32], [34] showed a negative correlation between sedentary time and LIPA (r=−0.96) in adults and an inverse linear relationship between LIPA and a number of cardio metabolic biomarkers, promoting LIPA seems to be an effective and feasible approach to decrease the negative health consequences of SB in adults. Similar studies in youth, however, are still lacking. Based on the latter studies, Hamilton et al. [35] suggest to promote a healthy balance between time spent in LIPA and SB (i.e., spending more time in LIPA than in SB). To the best of our knowledge, no studies have reported prevalence rates of the LIPA-SB balance yet.

The present study aimed to get insight into the prevalence rates of objectively measured sedentary time and LIPA in the Belgian population. Parallel to the PA literature it is also important to look at prevalence rates of SB and LIPA *across the lifespan.* As there is emerging evidence that SB is a risk behavior in adults , it is of interest to know if the prevalence of SB during the lifespan increases as MVPA decreases or whether another type of relationship exists and if this relationship is the same in men and women. Therefore, we examined whether the amount of time spent in SB, LIPA and MVPA differed across several age groups (preschoolers, primary schoolchildren, secondary schoolchildren, and adults) and between men and women. Further, following the recommendations of Hamilton et al. [35], a second aim of the present study was to explore which proportion of the population had a favorable balance between LIPA and SB (i.e., time spent in LIPA > SB) and whether or not a positive balance was combined with sufficient MVPA.

## Methods

### Participants and procedure

Accelerometer data from seven previous studies [36], [37], [38], [39], [40] in four different age groups (preschoolers, primary schoolchildren, secondary schoolchildren and adults) were made available for secondary data analysis. All studies took place in Flanders, Northern part of Belgium, between September 2003 and April 2010. Detailed description of the design and sampling procedures for each study are reported elsewhere [36-40].

In short, *study l* was a cross-sectional study [36], in which 261 participants (3–6 years old) from 45 randomly selected preschools were involved. Children wore an accelerometer during six consecutive days, including two weekend days. During the first day, a researcher fitted the children with an accelerometer at preschool. Parents of the participating children were provided an instruction form to ensure correct accelerometer use. On day six, a researcher recollected all accelerometers at preschool.

*Study 2* was an intervention study in primary schoolchildren (10–12 years old) [37]. PA was objectively assessed during five consecutive days, including two weekend days, in a subsample of 123 children from eight randomly selected primary schools. On day one, during baseline measurements of the intervention study, a researcher at the school distributed the accelerometers. To ensure correct accelerometer use, an accelerometer instruction form was used to inform the parents of the participating children. After five days, accelerometers were recollected at school.

*Study 3* had a longitudinal design (unpublished data). In the baseline measurements of this study, a subsample of 304 children (10–13 years old) from 44 primary schools wore an accelerometer for seven consecutive days. On day one, a researcher at the school distributed the accelerometers. After seven days, accelerometers were recollected at school.

*Study 4* was an intervention study in secondary schoolchildren (11–15 years old) [38]. A subsample of 186 adolescents was randomly selected from 15 participating secondary schools to wear an accelerometer for six consecutive days, including two weekend days. On day one, during baseline measurements of the intervention study, a researcher at the school distributed the accelerometers. After six days, accelerometers were recollected at school*.*

In *study 5*, a cross-sectional study, a random sample of 637 adolescents (12–16 years old) were recruited from 32 different neighborhoods [41]. Participants were visited at home and asked to wear an accelerometer for seven consecutive days. After seven days, a researcher collected accelerometers during the second home visit.

In *study 6*, a reliability and validity study, a random sample of 60 adults (18–65 years) was selected in three different neighbourhoods [39]. Participants were visited at home and asked to wear an accelerometer for seven consecutive days. After seven days, a researcher collected accelerometers during a second home visit. For the secondary data analyses of the present study, accelerometer data of students (n=9) and retired adults (n=1) were excluded.

*Study 7* was a cross-sectional study in adults (18–65 years old) [40]. A random sample of 1066 adults in 24 neighborhoods was visited at home and asked to wear an accelerometer for seven consecutive days. After 7 days, a researcher recollected the accelerometers during the second home visit. For the secondary data analyses of the present study, accelerometer data of students (n=37) and retired adults (n=98) were not included.

The Ethics Committee of the Ghent University Hospital (UZ Ghent) approved all studies and the participants or their parents gave written informed consent, if participants were younger than 18 years old.

### Anthropometry

In the first five studies, collecting data in children and adolescents, participants’ height was measured objectively to the nearest 0.1cm with a portable stadiometer (SECA model 214) and participants’ weight was measured to the nearest 0.1 kg with a beam balance scale (Model 813; SECA, Hamburg, Germany). In studies 6 and 7, the two adult studies, participants’ height and weight were self-reported.

Height and weight were used to calculate BMI (kg/m^2^). Based on the age- and gender-specific BMI cut-offs of Cole et al. [42] participants were categorised as normal weight, overweight or obese. Finally, BMI z-scores were calculated using Belgian reference data [43].

### Assessment of SB and PA

To objectively assess SB and PA, the ActiGraph accelerometer model 7164 and ActiGraph GT1M (Manufacturing Technologies Inc., Shalimar, FL) were used in the included studies. Both types are uniaxial accelerometers, designed to detect vertical accelerations, and studies have shown that data collected from both types are comparable to each other for estimating habitual activity levels and can be used in the same study [44], [45]. It has been shown that the Actigraph accelerometer is a valid, reliable and objective method for monitoring PA in preschoolers [46], children and adolescents [47-49] and adults [50]. In all studies, the accelerometers were initialized to save data over 60 seconds epoch time intervals, except for study 1 [36] in which a 15 seconds epoch time interval was used to capture the spontaneous PA of preschoolers [51,52]. In all studies, the accelerometer was worn on the right hip and was held in place by an adjustable elastic belt. Participants were instructed to wear the accelerometer during waking hours and to remove the accelerometer only for sleeping, water-based activities such as bathing or swimming and activities that prohibit an accelerometer (e.g. judo).

### Data reduction

Accelerometer data were scored and interpreted using the MeterPlus Version 4.2 software from Santech. Inc. (http://www.santechhealth.com). The first day on which the accelerometer was delivered by the researcher and the last day on which the accelerometer was recollected were omitted from the data file because of incompleteness. Participants were included in the analysis if the minimal number of accelerometer wearing days was 3 (with at least one weekend day) and if minimal number of wearing time was 10 hours per weekday and 8 hours per weekend day [51]. Non-wearing time was defined as 60 minutes or more of consecutive zero counts.

Due to a lack of consensus about the cut points, a wide range of cut points is identified in the literature to calculate sedentary time, or time spent in LIPA and in MVPA (e.g., thresholds range from 190 cpm [53] in adults to 3561 cpm [54] in 5 year olds). As there is no consensus in the literature whether age-specific cut points are needed in youth, we selected cut-points that are reported in the literature in both youth and adults. We used a cut-point of < 100 cpm (or < 25 counts/15 seconds for preschoolers) to calculate time spent in SB, a range of 100–2000 cpm (or 25–500 counts/15 seconds in preschoolers) for LIPA and > 2000 cpm (or >500 counts/15 seconds in preschoolers) for MVPA. The 100 cpm cut-point value for sedentary time has been reported in calibration studies in 5 to 15 year-olds [55], [56] and in a small validation study in adults [29]. Moreover, this cut-point has also been used in several studies to explore the relationship between SB and health [32,34], [57] or to identify prevalence rates of SB in large population samples [29-31,33]. The cut-point value of 2000 cpm, corresponds with a walking speed of 3–4 km/h [58,59] and has been used in previous studies in children and adults to categorise MVPA [33], [20,60-62]. Values above 16,000 cpm were considered as an equipment malfunction and excluded as they are beyond the plausible range of human movement. Accelerometer data with more than 10 minutes of malfunction were omitted from the data file.

### Data analyzing

Only participants with complete accelerometer and anthropometric data were included in the secondary data analyses (N=554 were excluded). This resulted in a total sample of 2083 participants (207 preschoolers, 348 primary schoolchildren, 587 secondary schoolchildren and 940 adults).

Accelerometer data collected with a 15 s epoch interval (study 1) were adjusted (multiplied by 4) so that all values of non-wearing time, sedentary time, time spent in LIPA and in MVPA were expressed as minutes. Values of total counts, non-wearing time, time spent in SB, LIPA and MVPA were averaged for all valid days (counts/day or min/day). Total wearing time was calculated by subtracting non-wearing time from the total observation time for the day (1440 minutes or 24 hours). Total PA level (expressed in cpm) was calculated by dividing the average number of total counts per day by monitor-wearing time in minutes. Total time spent in SB, LIPA and MVPA per day was also expressed as a proportion (percent) of monitor-wearing time.

Average time spent in MVPA per day was used to calculate whether participants fulfilled the guidelines of PA, namely 60 minutes of MVPA per day in children and adolescents [63] and 30 minutes of MVPA per day for adults [7].

Based on the recommendations of Hamilton et al. [35] the balance between percentage of time spent in LIPA and percentage of time spent in SB was calculated (LIPA minus SB). A positive balance indicates more time spent in LIPA than in SB. In addition, participants were divided in four categories based on whether they did sufficiently or insufficiently MVPA using age relevant recommendations [7,64-66] and whether they had a positive or negative balance between LIPA and SB. In the first category, participants fulfilled the MVPA guidelines and had a positive LIPA-SB balance. The second category contained the participants who performed sufficient amounts of MVPA, but had a negative LIPA-SB balance (also called the Active couch potato phenomenon [24]). Participants in the third category did not fulfil the MVPA guidelines but had a positive LIPA-SB balance. Finally, the fourth, unhealthiest, category included participants who did not perform sufficient amounts of MVPA and spent too much of their time in SB (negative balance).

### Statistical methods

All variables were checked for normality by means of the Kolmogorov-Smirnov test. Due to positive skewness of total PA level, time in SB, time in LIPA, time in MVPA and percent time in MVPA, analyses were executed using log transformed data [67] for these variables. In the descriptive tables and figures, non-transformed data are presented.

Differences in SB and PA between age groups and between men and women were determined by two-way MANCOVA with SB and PA behaviors (total PA level, percent time in LIPA and percent time in MVPA) as dependent variables, age groups (preschoolers, primary schoolchildren, secondary schoolchildren, adults) and gender as the independent variables. BMI-z-score was included as a covariate in the analysis as it is related to the dependent variables (SB, PA).

If relevant, in depth analyses, two-way MANCOVA comparing two age groups, were performed. Pearson’s correlations coefficients were used to examine the associations between SB and all PA measures All analyses were performed using SPSS 15.0 software (SPSS Inc., Chicago, IL, USA) and the level of significance was set at p<0.05.

## Results

### Characteristics of participants

Characteristics of the total sample, stratified by gender and age group, are described in Table 1. The total sample consisted of 2083 participants of which 52% women. Seventy-nine percent of the total sample was classified as normal weight, 17% as overweight and 4% as obese. BMI of the adult group of our study (24.2 kg/m^2^) was significantly lower compared with that of the Belgian adult population (24.8 kg/m^2^) [68]. Other characteristics were similar with characteristics of the Belgian population.

**Table 1:** Characteristics of the sample stratified by gender and age groups

**Men + women**	**Preschool (study 1)**	**Primary school (study 2,3)**	**Secondary school (study 4, 5)**	**Adults (study 6, 7)**	**All age groups**^**1**^
**N**	207	348	587	941	2083
**Age (years)**					
**Mean (SD)**	4.5 (0.8)	10.7 (1.0)	13.6 (1.2)	41.8 (11.2)	17.7 (15.5)
**Range**	3-6	8-13	11-16	19-64	3-64
**BMI (kg/m**^**2**^**)**	15.8 (1.4)	17.7 (2.5)	20.2 (3.6)	24.3 (3.9)	19.5 (4.4)
**Normal weight**	85%	85%	81%	64%	79%
**Overweight**	14%	13%	15%	28%	17%
**Obese**	1%	2%	4%	8%	4%
**Men**	**Preschool**	**Primary school**	**Secondary school**	**Adults**	**All men**^**1**^
**N**	114	171	280	441	1006
**Age (years)**					
**Mean (SD)**	4.5 (0.8)	10.9 (1.0)	13.7 (1.2)	41.5 (11.0)	17.1 (15.1)
**Range**	3-6	8-13	11-16	20-63	3-63
**BMI (kg/m**^**2**^**)**	15.9 (1.3)	17.6 (2.3)	20.0 (3.5)	25.3 (3.7)	19.5 (4.6)
**Normal weight**	87%	87%	82%	53%	78%
**Overweight**	12%	12%	15%	36%	18%
**Obese**	1%	1%	3%	11%	4%
**Women**	**Preschool**	**Primary school**	**Secondary school**	**Adults**	**All women**^**1**^
**N**	93	177	307	500	1077
**Age (years)**					
**Mean (SD)**	4.4 (0.8)	10.6 (1.1)	13.5 (1.2)	42.1 (11.4)	18.3 (15.8)
**Range**	3-5	8-13	11-16	19-64	3-64
**BMI (kg/m**^**2**^**)**	15.8 (1.5)	17.8 (2.7)	20.5 (3.7)	23.3 (3.8)	19.5 (4.2)
**Normal weight**	82%	84%	81%	73%	80%
**Overweight**	17%	13%	14%	21%	16%
**Obese**	1%	3%	5%	6%	4%

### Time spent in SB and PA according to age groups and gender

Significant age by gender interaction effects were found for both SB and PA (Multivariate F age*gender=6.57, p< 0.001). Therefore, accelerometer data are presented for the total sample stratified by gender and age groups (Table 2). The average number of days measured for the total sample was 5.70 (±1.32) days, ranging from 3.89 (±0.31) days in preschoolers to 6.73 (±0.60) days in adults. Participants wore the accelerometer on average 13.30 (±1.69) hours a day, ranging from 12.12 (±1.61) hours in preschoolers to 14.54 (±1.52) hours in adults.

**Table 2:** Objectively measured SB and PA stratified by gender and age group.

**All**	**Preschool**	**Primary school**	**Secondary school**	**Adults**	**All age groups**^**1**^	**F**_age*gender_	**F**_**age**_
N	207	348	587	941	2083	MultivariateF=6.57**	Multivariate F=63.18**
**# days measured**	3.9 (0.3)	5.8 (0.9)	6.4 (0.9)	6.7 (0.6)	5.7 (1.3)		
**Wearing time (hours)**	12.1 (1.6)	12.9 (1.2)	13.6 (1.4)	14.5 (1.5)	13.3 (1.7)		
**SB (% wearing time)**	51 (7)	53 (8)	59 (9)	57 (11)	55 (9)	14.57**	57.74**
**LIPA (% wearing time)**	41 (5)	41 (7)	35 (8)	39 (10)	39 (8)	12.18**	42.27**
**MVPA (% wearing time)**	8 (3)	6 (3)	6 (3)	4 (3)	6 (3)	2.93*	159.60**
**Total PA (cpm)**	566(133)	462 (147)	416 (145)	365 (143)	452 (160)	9.13**	114.33**
**LIPA > SB (% of sample)**	21	22	8	20	18		
**Sufficient MVPA (% of sample)**	48	26	24	48	27		
**Men**	Preschool	Primary school	Secondary school	Adults	All men^1^		F _age_
**N**	114	171	280	441	1006		Multivariate F=26.74**
**# days measured**	3.9 (0.4)	5.8 (0.9)	6.4 (0.9)	6.7 (0.6)	5.6 (1.4)		
**Wearing time (hours)**	12.2 (1.9)	12.9 (1.3)	13.7 (1.4)	14.7 (1.5)	13.3 (1.8)		
**SB (% wearing time)**	51 (7)	52 (8)	57 (9)	59 (11)	55 (10)		28.62**
**LIPA (% wearing time)**	40 (6)	41 (7)	36 (8)	37 (10)	38 (8)		15.52**
**MVPA (% wearing time)**	9 (3)	7 (3)	7 (3)	4 (3)	7 (4)		74.81**
**Total PA (cpm)**	583 (147)	490(146)	474 (160)	381 (165.)	486 (170.)		51.21**
**LIPA > SB (% of sample)**	21	20	10	17	17		
**Sufficient MVPA (% of sample)**	56	36	39	56	47		
**Women**	Preschool	Primary school	Secondary school	Adults	All women^1^		F _age_
**N**	93	177	307	500	1077		Multivariate F=45.37**
**# days measured**	4.0 (0.2)	5.8 (1.0)	6.3 (1.0)	6.7 (0.6)	5.8 (1.3)		
**Wearing time (hours)**	12.0 (1.2)	13.0 (1.1)	13.5 (1.4)	14.4 (1.5)	13.3 (1.6)		
**SB (% wearing time)**	51 (6)	53 (8)	61 (9)	56 (10)	55 (9)		42.58**
**LIPA (% wearing time)**	41 (5)	42 (7)	35 (8)	41 (10)	40 (8)		40.15**
**MVPA (% wearing time)**	8 (2)	5 (2)	5 (2)	3 (2)	5 (3)		80.61**
**Total PA (cpm)**	546 (113)	435 (143)	362 (106)	351 (120)	418 (143)		64.27**
L**IPA >SB (% of sample)**	20	23	6	24	18		
**Sufficient MVPA (% of sample)**	38	15	10	41	26		

**Figure 1:**
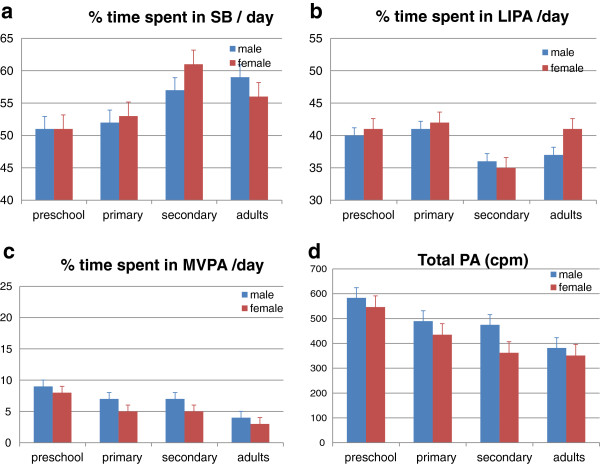
**Time spent in sedentary and physical activity behaviors according to age and gender.** * significant age x gender effect between two age groups (corrected for BMI z-score). men:  women.

On average, participants spent 55% of their time in SB, 39% in LIPA and only 6% in MVPA. In Figure 1 (a-d), the percentages of time spent in SB, LIPA and MVPA are shown stratified by gender and age groups.

Figure 1a shows the time spent in SB for each age group. Results showed that differences between the age groups were gender dependent (F age*gender=14.57, p<0.001). In boys, higher percentages in SB were found with increasing age (F age=28.617, p<0.001), with a minimum amount of 51% in preschoolers and a maximum amount of 59% in adults. In depth analyses showed significant difference between all age groups (p<0.05) except between the two youngest age groups (p=0.153). In girls, another pattern was found. The maximum amount of SB was measured in secondary schoolgirls (61%), who spent significantly more time in SB compared to the younger (51% and 53% for preschoolers and primary schoolchildren respectively) and oldest (56% in adults) age groups (p<0.001).

Figure 1b shows the time spent in *LIPA* for the different age groups and gender (F age * gender = 42.270, p<0.001). In depth analyses showed no significant age by gender differences in LIPA between the two youngest age groups. Secondary school children, however, performed less LIPA compared to primary schoolchildren and this difference was significantly more obvious in girls (−7.04%, p<0.001) than in boys (−4.51%, p<0.001). Adults on their turn spent more time in LIPA than adolescents, however this difference was only significant in women (+5.84%, p<0.001) and not in men (+0.61%, p=0.643).

Time spent in *MVPA* in the different age groups and gender is shown in Figure 1c. There was a linear trend (F age= 159.603, p<0.001) showing older age groups spending less time in MVPA, however no significant differences were found in MVPA between primary and secondary schoolchildren. In all age groups there was a similar pattern in men and women, however, the difference between preschool and primary schoolchildren was significantly larger in girls (−2.66%) than in boys (−1.98%) (F age*gender=6.484; p=0.011).

Total PA level, expressed in cpm, in the different age groups and gender is illustrated in Figure 1d. Similar as with time spent in MVPA, total PA level was lower in the older age groups (F age=114.33, p<0.001). Again, both men and women had a similar pattern across the lifespan, however, the difference between primary and secondary schoolchildren was only significant in girls (−73 cpm, p<0.001) and not in boys (−15 cpm, p=0.808) (F age*gender=9.489, p=0.002).

### Correlations between SB and PA

Significant negative Pearson’s correlations were found between time spent in SB and all PA measures (% LIPA, % MVPA, total PA). For the total sample (N=2083), the strongest correlation was found between time spent in SB and time spent in LIPA (weighted r_p_ =−0.9, p<0.001). Time spent in SB was also significantly negatively correlated with total PA (weighted r_p_=−0.8, p<0.001) and with time spent in MVPA (weighted r_p_=−0.5, p<0.001).

### Balance between LIPA and SB

More LIPA than SB was found in 18% of the total sample. Thirty-eight percent of the sample fulfilled the guidelines of MVPA (60 minutes MVPA per day for children and 30 minutes MVPA per day for adults).

**Figure 2:**
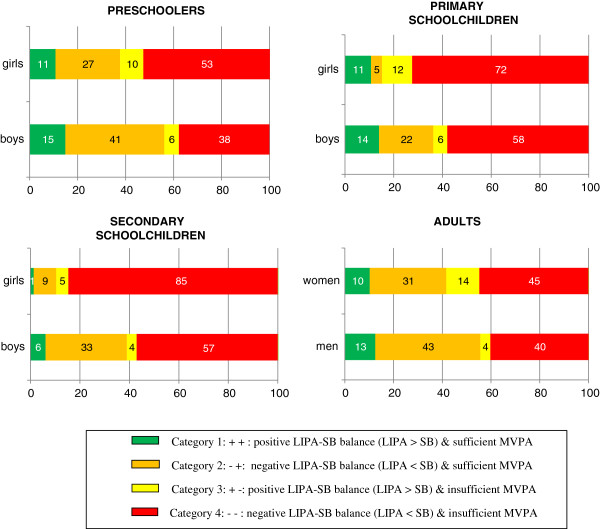
Percentage of the sample combining a (un)healthy LIPA-SB balance with (in)sufficient MVPA.

Figure 2 shows the proportions of participants, according to age groups and gender, in the following four categories (1) positive LIPA-SB balance (LIPA> SB) and sufficient MVPA (2) negative LIPA-SB balance and sufficient MVPA (3) positive LIPA-SB balance and insufficient MVPA (4) negative LIPA-SB balance and insufficient MVPA. Only a small proportion of the total sample (10%) was classified in the first category, indicating that they had a positive LIPA-SB balance (LIPA > SB) and fulfilled the guidelines of MVPA. The proportions ranged from 1% in secondary schoolgirls to 15% in preschooler boys.

A second category included the participants (26% of total sample) who had a negative LIPA-SB balance (LIPA < SB) but did sufficient MVPA. The smallest proportion in this category was found in primary schoolgirls (5%) and the largest in adult men (43%).

A third category included 8% of the total sample who had a positive LIPA-SB balance, but did not fulfil the MVPA guidelines. The minimum proportion was found in secondary schoolboys (4%) and the maximum proportion in adult women (14%).

Finally, 56% of all participants were categorised in the fourth category. Those participants spent more time in SB than in LIPA (negative LIPA-SB balance) and did not do sufficient MVPA. The smallest proportion of this category was found in preschooler boys (38%), the largest proportion in secondary schoolgirls (85%).

## Discussion

The aims of this study were to objectively investigate the time spent in SB and PA in a lifespan perspective by investigating accelerometer data in different age groups (preschoolers, primary schoolchildren, secondary schoolchildren and adult) and to calculate the balance between time spent in LIPA and SB.

The first finding of the present study was that Belgian preschoolers, primary and secondary school children, and adults (3–65 years old) spent on average 55% of their waking time (7.3 hours) in SB (range 51%-59% for different age groups), which is equal to the prevalence rate found in the US population (6–85 years old) [29]. The average time spent in LIPA (39% or 2.9 hours a day) and MVPA (6% or 45 minutes a day) found in the Belgian sample was difficult to compare with other accelerometer studies as different age ranges, cut-off points or data reduction rules were used. Nevertheless, our results confirmed that sedentary time constitutes the bulk of waking hours in Western countries, with the remainder of time disproportionately spent in LIPA and MVPA. Sitting time is therefore an important target behavior in health behavior changes programs.

A second important finding of our study was that differences in sedentary time and PA levels between age groups were gender dependent. Our results revealed a linear relationship in men, namely higher percentages in SB (and lower percentages in PA) with increasing age, resulting in a maximum amount of 59% of SB in adults. However, in women a reversed U curve was found, identifying secondary schoolchildren as the most sedentary group with 61% of their waking time being sedentary. This is in line with the findings of Matthews et al. [29] who reported a peak of sedentary time (60%) in US adolescents (16–19 years old). However, in contrast with the current study, Matthews et al. found also a reversed U-curve in adolescent boys, who spent more time in SB compared to adults under 50 years old.

Due to the cross-sectional study design it is unclear whether the age-related gender effects are influenced by developmental changes or whether cohort effects are present. Hypothesizing that cohort effects are minimal between two consecutive age groups, it could be assumed that secondary schoolchildren (when compared to primary schoolchildren) replace LIPA by SB, whereas percentage of time spent in MVPA is still very low (6%). In adulthood (when compared to secondary schoolchildren), women reverse the pattern by decreasing their sedentary time and increase their PA levels, but only at light intensity. The latter showed that high prevalence rates of sedentary time found in secondary schoolgirls are partly restored by itself during adulthood. This remarkable substitution of SB into LIPA in adult women is probably associated with an increase in time spent doing household chores and child care, activities mostly done at light intensity [29]. In contrast, our data reveal that adult men become another important risk group as their amount of SB increases with increasing age. They probably replace their sports activities (MVPA) during adolescence by SB in adulthood. Large longitudinal studies with objective assessments, supplemented by context-specific self-reports, are needed to confirm these hypotheses and exclude potential cohort effects.

The finding that Belgian secondary schoolgirls spent more time in SB compared to secondary schoolboys is in accordance with the accelerometer data found in US youth [29], but in contrast to a previous European study using self-report data in adolescents [69]. This contradiction stresses the importance of accurate methods to measure SB as it seems that self-reported screen time behaviors do not capture overall sedentary time, especially in adolescent girls [27,70,71].

A third important finding of our study is the low prevalence rate of participants with a positive LIPA-SB balance, namely 18% of the total sample. Based on the studies of Healy et al. [32], Hamilton et al. [35] suggested that a positive balance between time spent in LIPA and SB might be health beneficial in adults To our knowledge, no prevalence data for this balance have been reported in the literature yet. Our results showed that the smallest proportion (6%) of a positive LIPA-SB balance was found in secondary schoolgirls. Recent research has proven the independent effect of SB on health in adults [12], [13], [14],[15], [16], [17], [18], [22], [23] which means that, to profit from the health benefits, people should 1) fulfill the PA guidelines and 2) limit their time spent in SB. In our Belgian sample, 36% fulfilled the PA guidelines, but only 10% combined sufficient PA with a positive LIPA-SB balance. In secondary schoolgirls, only 1% combined a positive LIPA-SB balance with sufficient MVPA each day. Also remarkable is the high proportion of adult men (43%) who are combining sufficient MVPA with a negative LIPA-SB balance. Those men are probably not aware of their increased health risk caused by high levels of sedentary time, as SB was not included in the PA recommendations promoted during the last decennia. Although the present study has revealed that there are groups of ‘active men’ that also have a negative LIPA-SB balance, it should be further investigated if this LIPA-SB balance is indeed a good health indicator in itself. Also the evidence for the negative health impact of SB and the positive health impact of LIPA is consistent in adults but until now mixed results are found in youth [21]. So more research on the health impact of SB, LIPA and balance LIPA-SB in the younger age groups is needed.

In PA literature, adolescent girls have been recognized as an important target group for interventions [72]. Based on the results of the current study, we recommend that next to promoting MVPA, the reduction in SB in this target group is very important as well. As adolescent girls were not identified as an at risk group for SB in studies using self-reports, it is possible that existing questionnaires insufficiently capture those types of sedentary activities adolescent girls engage in. Qualitative studies with adolescent girls can provide more in depth insight into the SB contexts.

Interventions aimed at decreasing SB and increasing PA, should therefore not only focus on MVPA but also on LIPA. The highly negative correlations between SB and LIPA found in our study (r=−0.94) and in the study of Healy et al. [34] (r=−0.96) indicate that replacing SB by LIPA is a good alternative to decrease time spent in SB in people who are not able or willing to increase their MVPA levels. Further studies should also determine which types of sedentary behaviors are most common in each age group and which behaviors are most feasible to replace by which types of LIPA.

### Strengths and limitations

This study objectively assessed SB in a large sample and is one of the first to report prevalence rates about the LIPA-SB balance as recommended by Hamilton et al. [35]. The results of our study can probably be generalized to the Belgian population, due to the sample size, different age groups and the probability sampling method.

However, this study also has several limitations. First, the study design was *cross-sectional* which comprises that age-effects could be confounded by cohort effects.

Secondly, accelerometer data were collected from different PA studies for this secondary data-analysis. One of the consequences was that a 15 seconds *epoch time* was used in the youngest age group, whereas a 1 minute epoch time was used in the other age groups. Data of the preschoolers were transformed to cpm, however Corder et al. [73] showed that a different epoch length affects the estimation of time spent in different activity categories. Another consequence was that we used the same PA guideline for preschoolers [66] as for primary and secondary schoolchildren (60 minutes MVPA/day). However, recent guidelines for preschoolers developed in the UK [65] and Australia [64] do not longer take into account the intensity of PA but recommend 3 hours a day of total PA.

A third limitation is that by using accelerometry only the amount of SB and PA could be collected but nothing is known about the *behavioral context*. Therefore, questionnaires that assess a wide range of sedentary and PA behaviors, remain important to gain additional information that is necessary to develop interventions.

Further, *cut-offs* were used to categorise activities as SB, LIPA or MVPA. In the literature, the 100cpm cut-off for SB is very common [29-34,55-57]. However, it has been shown that a hip-mounted accelerometer, measuring accelerations in the vertical plane, cannot correctly make the distinction between sitting and standing still [27]. According to the definition of The Sedentary Behavior Research Network [6], standing still is not categorised as SB but as LIPA because standing requires muscle contractions. Therefore, the amount of time spent in SB measured with accelerometers could somewhat be overestimated by incorporating periods of standing still, and as a consequence LIPA somewhat underestimated. Future studies could overcome this bias by taking into account posture, assessed for example by an inclinometer [74].

Also the cut-offs for LIPA and MVPA are often subject of discussion in the literature and differ between age groups. Therefore it is difficult to compare our PA results with other accelerometer studies using different cut-offs.

A final limitation of the study is that we did not take into account the *bouts* of both SB and PA. For SB, it has been shown [11] that not only the amount of SB (minutes a day) but also the breaks (prolonged sitting versus regular breaks) have an influence on health parameters. It was beyond the scope of this paper to make differences between “prolongers” and “breakers” but future research should take this into account. With respect to PA behavior, the guidelines of MVPA for adults include that minutes can be accumulated throughout the day, but in blocks of minimum 10 minutes. The bouts of PA were not analysed in our study and therefore the percentage of participants fulfilling the guidelines could even be lower than the percentage reported here. In the study of Hagstromer et al. [30] the proportion of the adult sample that fulfilled the PA guidelines assessed with an accelerometer decreased from 52% to 1% when only blocks of at least 10 minutes MVPA were included.

## Conclusion

Belgian children, adolescents and adults spent most of their waking time sedentary (55%). Different SB levels were shown between age groups according to gender. In women, a reversed U-shape was found, with girls at secondary school being the most sedentary (61%). In men, time spent in sedentary time increased with age (51% in preschoolers until 59% in adults).

Considering both SB as well as PA levels, a high proportion of the Belgian population (90%) is at risk, warranting interventions to decrease SB and increase LIPA and MVPA in all age groups. Again, secondary schoolgirls have the unhealthiest SB and PA profile and are therefore of particular interest for interventions. Also adult men are an important target group in which more attention should be given in promoting a positive LIPA-SB balance independently from their compliance with the MVPA guidelines.

## Abbreviations

SB: Sedentary Behavior; MVPA: Moderate-to-Vigorous Physical Activity; LIPA: Light Intensity Physical activity; PA: Physical Activity; BMI: Body Mass Index; US: United States; UK: United Kingdom; Cpm: Counts per minute.

## Competing interests

The authors declare that they have no competing interests.

## Authors’ contribution

HS, LH, BD, GC, and IDB identified the research question and design of this study. EVC, VV, MV, FDM, DVD and LH collected the data and helped with the data reduction. HS leaded the data reduction, did the statistical analysis and drafted the manuscript. All authors contributed to synthesising the results and critical revision of the manuscript, and approved the final version.
